# ﻿Taxonomic clarification of *Silenedawoensis* (Caryophyllaceae): synonymization with *S.batangensis* and reinstatement of *S.longiuscula*

**DOI:** 10.3897/phytokeys.257.153202

**Published:** 2025-06-03

**Authors:** Feng Yang, Huan-Chong Wang

**Affiliations:** 1 School of Ecology and Environmental Science, Yunnan University, Kunming 650091, China Yunnan University Kunming China; 2 Herbarium of Yunnan University, Kunming 650091, Yunnan, China Herbarium of Yunnan University Kunming China

**Keywords:** China, *Cucubaloides*, Jinshajiang Valley, sect, *Silene*, taxonomy

## Abstract

Based on a comprehensive analysis of type materials, protologues, herbarium specimens, and field observations, this study clarifies the taxonomic status of *Silenedawoensis* and related species in China. Our findings reveal that *S.dawoensis* shares key similarities with *S.batangensis*, including leaf shape, inflorescence type, and calyx structure, and therefore we have placed S.dawoensis as a synonym of S.batangensis. Additionally, specimens historically misidentified as *S.dawoensis* are reassigned to *S.markamensis*, a species previously overlooked in regional floras. Concurrently, based on diagnostic differences in leaf shape, calyx length, and petal morphology, we reinstate *S.longiuscula* as a distinct species, previously treated as a synonym of *S.dawoensis*. Typification for *S.batangensis* and *S.dawoensis* is given. Descriptions, distribution maps, taxonomic notes, and morphological comparison table are provided for these three species.

## ﻿Introduction

*Silene* L. is a large genus of the carnation family comprising 700 to 870 species, which mostly occur in temperate regions and subtropical mountains of the Northern Hemisphere (Aksoy et al. 2008; Mabberley 2017; Jafari et al. 2020). The Mediterranean area and Western Asia are two of the main diversity centers of *Silene*, but regions of Central Asia are also highly diverse (Jafari et al. 2020). Studies on the evolutionary history of *Silene* species have identified three main evolutionary lineages consisting of 3 subgenera and 35 sections (Eggens et al. 2020; Jafari et al. 2020). Many species of *Silene* have high horticultural, medicinal and ecological significance (Chandra and Rawat 2015; Zengin et al. 2018; Zhang et al. 2021). Furthermore, certain species are used as model systems in studies of ecological adaptation (Scha 1993), pollination ecology (Buide et al. 2015), evolutionary biology (Bernasconi et al. 2009), and sex determination genetics (Papadopulos et al. 2015).

*Silene* in China was recently revised by Zhou et al. (2001) for Flora of China, in which 110 species are recognized and 67 species are endemic. *Silene* taxa can be found throughout the country, mainly distributed in the Qinghai-Tibet Plateau, southwestern mountains and northern arid areas. With the continuous advancement of research, there have been reports of new species and new records being discovered in China in recent years (Lin et al. 2019; Yang et al. 2022a, 2022b, 2023; Liu et al. 2023; Lidén and Oxelman 2023).

During the preparation of the Caryophyllaceae volume for the Flora of Pan-Himalaya, we conducted a comprehensive study on *Silene* L. (Caryophyllaceae) in the Sino-Himalayan region, encompassing field investigations, critical literature reviews, and examinations of herbarium specimens. Preliminary findings from this work have been published in our previous contributions (Yang et al. 2022a, 2022b, 2023). In the present study, our focus is on resolving several taxonomic issues surrounding *Silenedawoensis* H. Limpr., particularly regarding its identity.

## ﻿Materials and methods

This study is primarily based on a combination of literature review, specimen examination and field observations. Relevant collections housed at A, BNU, CDBI, CSH, CQNM, E, HITBC, HNWP, IBSC, KUN, LBG, NAS, PE, SM, WUK, XTBG, and YUKU (herbarium abbreviations according to Thiers (2025, continuously updated) were extensively reviewed and compared. In addition, digital images from online databases, including the Chinese Virtual Herbarium (CVH; https://www.cvh.ac.cn/), the Integrated Digitized Biocollections (iDigBio; https://www.idigbio.org/), the Global Biodiversity Information Facility (GBIF; https://www.gbif.org/), and the JSTOR Global Plants database (https://plants.jstor.org/), were also examined. Fieldwork was conducted in their natural range, including the Hengduan Mountains and Jinshajiang River Valley in southwest China. The main focus of the field observations was to document features not easily observed in dried specimens, such as floral structure, growth habits, and variation of key taxonomic characters in their natural habitats. The dot-distribution map was compiled from all specimens studied and generated with ArcGIS version 10.4 (ESRI, Inc., Redlands, USA). To further analyze the directional trends of distribution, the standard deviation ellipse tool in ArcGIS was used to create secondary standard deviation ellipses for the distribution points of the three species.

## ﻿Results and discussion

In the Flora Reipublicae Popularis Sinicae (FRPS), *Silenedawoensis* H. Limpr. is recognized as a perennial species with no taxonomic synonyms. Its distribution is restricted to western Sichuan and northwestern Yunnan, China. Tang (1996) classified this species within S.sect.Longitubulosae C. L. Tang, which according to Jafari et al. (2020) is a synonym of S.sect.Siphonomorpha Otth, and emphasized its morphological affinity to *S.esquamata* W.W. Sm. Additionally, *S.batangensis* H. Limpr. is treated as a member of S.sect.Cucubaloides Edgew. et Hook. f., occurring in western Sichuan and eastern Xizang (Tang 1996). In the subsequently published Flora of China, Zhou et al. (2001) has treated *S.longiuscula* C. Y. Wu et C. L. Tang as a new synonym of *S.dawoensis.* However, they note that the type specimen of *S.longiuscula* differs from *S.dawoensis* in having a glandular-hairy calyx.

**Figure 1.**
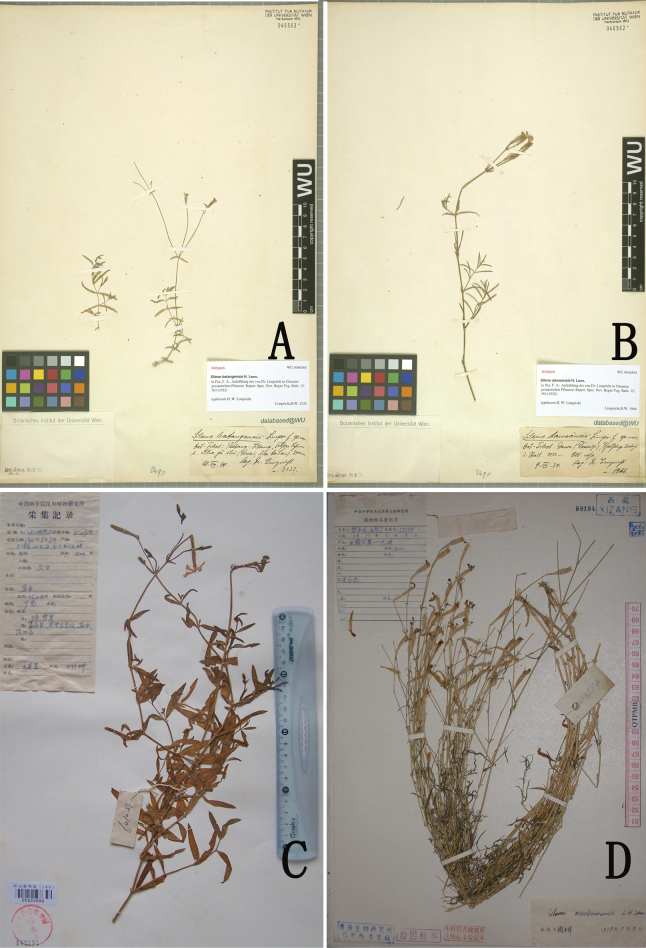
A comparison of the type specimens of *Silenebatangensis* (**A**), *S.dawoensis* (**B**), *S.longiuscula* (**C**), and *S.markamensis* (**D**).

**Figure 2.**
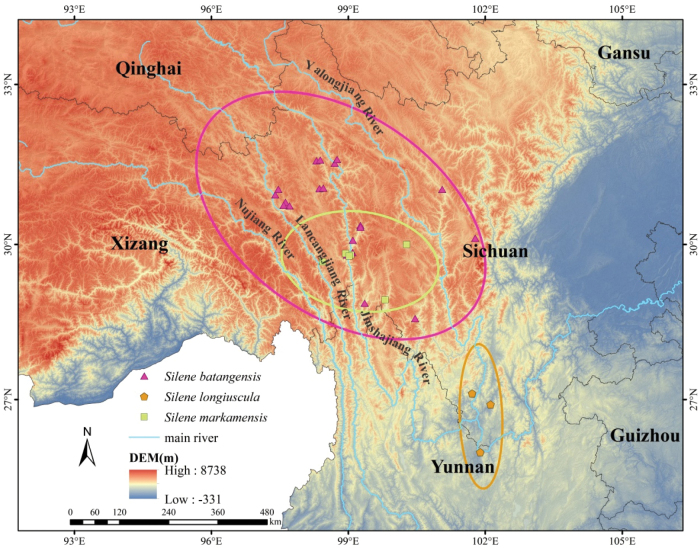
Distribution of *Silenebatangensis*, *S.longiuscula* and *S.markamensis* in southwest China.

**Figure 3.**
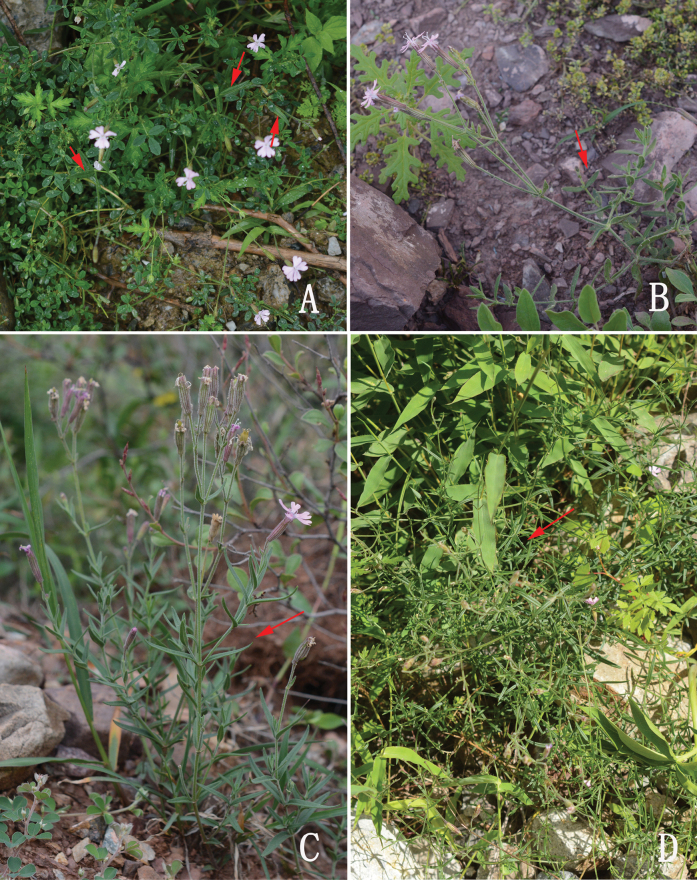
*Silenebatangensis* from different populations **A** Songduo Country, Batang, Sichaun, *F. Yang et al. BT22486* (YUKU) **B** near Big footprints, along the Langcang River, Chaya, Xizang, *F. Yang et al. CY22880* (YUKU) **C** near Rumei village, along the Lancang River, Mangkang, Xizang, *F. Yang et al. MK22969* (YUKU) **D** Zhubalong village, Mangkang, Xizang, *F. Yang et al. MK26730* (YUKU). Noting that the plants in A has narrowly lanceolate, glabrous leaves, B has lanceolate leaves, with densely glandular hairs at margins, C has narrowly lanceolate leaves, with densely glandular hairs at margins, D has linear, glabrous leaves.

*Silenedawoensis* and *S.batangensis* were published at the same time by Limpricht (1922) in the “Botanische Reisen in den Hochgebirgen Chinas und Ost-Tibets”. In the protologue, Limpricht (1922) cited one collection “Ost-Tibet: Dawo, Talhange unterbalb der Stadt, 3450 m (*n. 1966*)” for *S.dawoensis*, and “Ost-Tibet: Batang, felsige Lehnen bei Mba ju tschi, am Wege nach Litang, 3400 m (*n. 2232*)” for *S.batangensis*. We traced one sheet of *S.dawoensis* deposited in WU (barcode: WU0046562 digital image!) (Fig. 1A), and one sheet of *S.batangensis* (barcode: WU0046563 digital image!) (Fig. 1B). After carefully comparing the two specimens (Fig. 1A, B), we found a handwritten identification signature in the lower right corner, with handwritten collection information. It can be confirmed that these two specimens were cited by Limpricht in the protologue for *S.dawoensis* and *S.batangensis* respectively. Our critical examination of type materials reveals that they have almost identical morphological characteristics, with no substantial differences in habits, indumentum, leaves, dichotomous cymes, tubular calyx, and petals. Therefore, these two taxa belong to the same taxonomic entity. According to our survey of specimens and living plants, furthermore, it is confirmed that the *S.batangensis* actually has many variable characteristics in leaf shape (linear, lanceolate, or narrowly lanceolate), indumentum (glabrous, pubescence or glandular) and pedicel length (Figs 2, 3). Given that *S.dawoensis* had long been misapplied to a species with lignified roots, lax thyrse, 2.5–3.5 cm tubular calyx (see next paragraph for detailed discussion), we treat *S.dawoensis* as a new synonym of *S.batangensis*.

After examination of the type and description in the protologue of *S.dawoensis*, it is shown that it has linear leaves, 1.5–2.5 cm long, dichotomous cymes, ca. 1.4 cm long calyx. We discovered that specimens previously identified as *S.dawoensis* do not match the original description and type, including *F.T.Wang 21682* housed at KUN (barcode: KUN 0513666), which serves as the voucher specimen for the *S.dawoensis* illustration in “Flora Reipublicae Popularis Sinicae”. These misidentified specimens have lignified roots, lax thyrse, cymules opposite or alternate, 2.5–3.0 cm long tubular calyx, reniform, slightly flat seeds with a groove along the seed ridge, forming a shallow “V” shape. Further research revealed that these specimens previously identified as *S.dawoensis* actually belong to *S.markamensis* L. H. Zhou, a name that had been overlooked by other botanists. *Silenemarkamensis* is a glabrous perennial herb, lignified roots, many branches at the base of the stems, linear or lanceolate leaves, lax thyrse, tubular calyx, clavate in fruit, 2.5–3.5 cm long, ca. 3 cm long petals, shallowly bifid (Fig. 5, Zhou 1983). The species is distributed in the upper reaches of the Jinshajiang River in eastern Xizang and western Sichuan. Therefore, the misidentification of *S.markamensis* is now clarified. According to Jafari et al. (2020) treatment, it should belong to S.sect.Siphonomorpha based on these morphological characters such as thyrsoid inflorescence, tubular calyx, and clavate in fruit.

**Figure 4.**
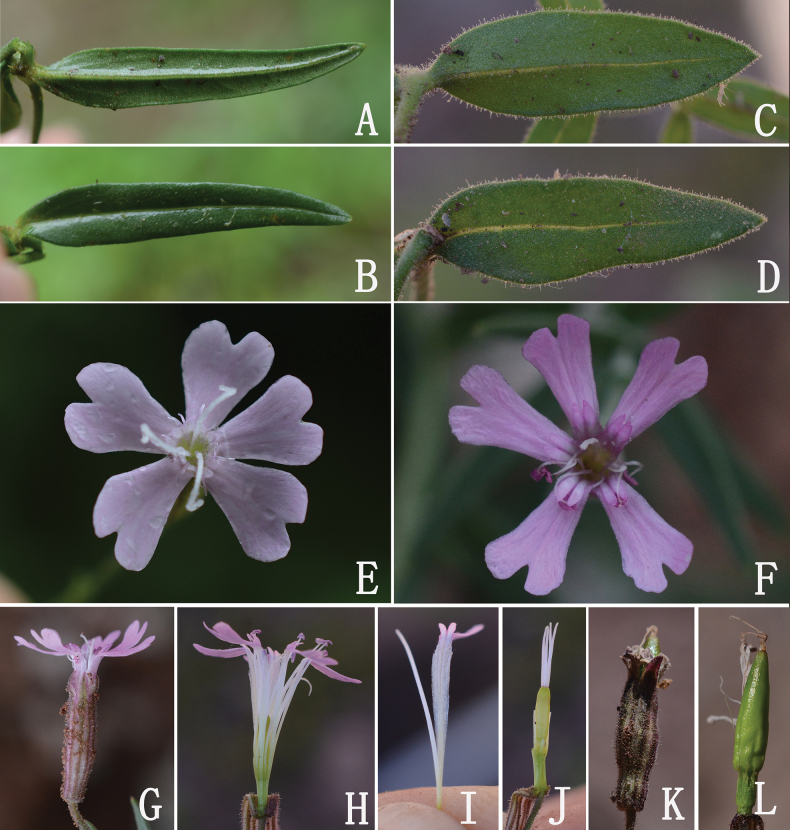
*Silenebatangensis* Limpric **A** abaxial surface of glabrous leaf from Songduo population **B** adaxial surface of glabrous leaf from Songduo population **C** abaxial surface of leaf from Rumei population, with densely glandular hairs at margins **D** adaxial surface of leaf from Rumei population **E** and **F** flowers (front view, showing the petals) **G** flower (side view, showing the calyx) **H** dissected flower (showing the androgynophore and claws) **I** petal (showing the claw, auricles and coronal scales) **J** pistil **K** calyx after anthesis **L** immature capsule.

*Silenelongiuscula* C. Y. Wu et C. L. Tang, was published by Wu and Tang in “Flora Yunnanica” based on the specimens collected in Yuanmou County (“Woody oilseed Team 65-709”) (Zhuang 1995), located in the middle reaches of the Jinshajiang River Valley. This name was not included in “Flora Reipublicae Popularis Sinicae” (Tang 1996) and was treated as a synonym of *S.dawoensis* in “Flora of China” (Zhou et al. 2001). After checking the original descriptions and types of the two species, we concluded that *S.longiuscula* and *S.dawoensis* can readily be distinguished from each other by the leaves (lanceolate, narrowly lanceolate or narrowly elliptic in *S.longiuscula* vs. linear, lanceolate or narrowly lanceolate in *S.dawoensis*), calyx (tubular vs. tubular-clavate, 3.0–3.3 vs. 1.2–1.5 cm long), and petals (narrowly obovate-oblong vs. obovate). It is not reasonable to treat *S.longiuscula* as a synonym of *S.dawoensis*. In addition, there are obvious differences between *S.longiuscula* and *S.markamensis* in the indumentum of the stems (downward white pubescent in *S.longiuscula* vs. glabrous in *S.markamensis*), leaf shape (lanceolate, narrowly lanceolate or narrowly elliptic vs. linear or linear-lanceolate), inflorescence (cymes with 3–15 flowers vs. few-flowered thyrse) (Figs 1C, D, 4). Table 1 summarizes a detailed morphological comparison among *S.longiuscula*, *S.markamensis* and *S.dawoensis* (synonymized with *S.batangensis*). Furthermore, we found that the distribution ranges of the two species do not overlap (Fig. 2). Therefore, it is worth recognizing *S.longiuscula* as an independent species. *Silenelongiuscula* and *S.batangensis* both belong to S.sect.Cucubaloides Edgeworth et Hook. f. since they have creeping habit, dichasial cyme and calyx tubular. *Silenelongiuscula* may represent a rare species, so rare that we have not found its wild population in many field surveys. However, in addition to the type specimen, we found three additional specimens during our specimen review.

**Figure 5.**
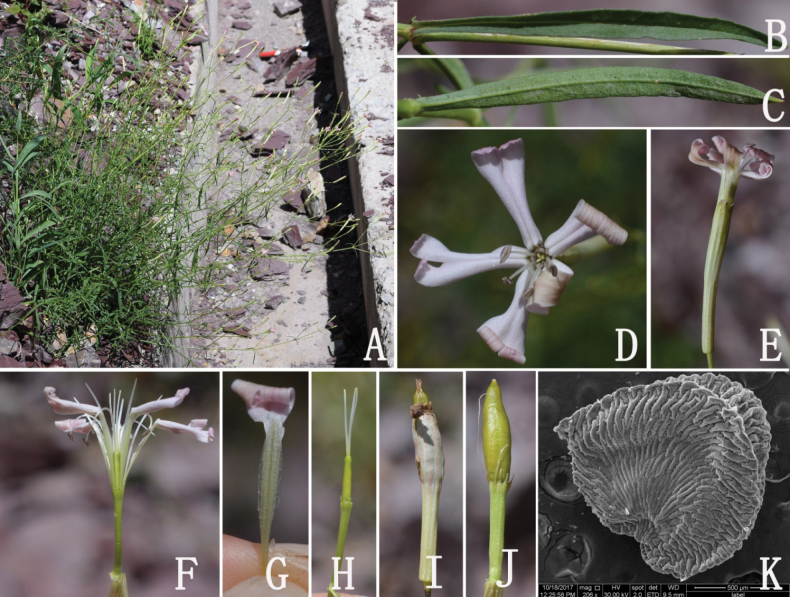
*Silenemarkamensis* L. H. Zhou. **A** habit **B** adaxial surface of the leaf **C** abaxial surface of the leaf **D** flower (front view, showing the petals) **E** flower (side view, showing the calyx) **F** dissected flower (showing the claws and androgynophore) **G** petal claw **H** pistil (showing the androgynophore, ovary and styles) **I** calyx after anthesis **J** immature capsule **K** seed (voucher specimens: *Sichuan Vegetation Team 3567*, deposited at the Herbarium of Kunming Institute of Botany (KUN)).

**Table 1.** Morphological comparison of *Silenebatangensis*, *S.longiuscula and S.markamensis*.

Characters	Species		
	*Silenebatangensis*	*Silenelongiuscula*	*Silenemarkamensis*
**Roots**	Clustered, fusiform	Not seen	Robust, conical, lignified
**Stems**	Supine or ascending, 20–35 cm long, multibranched, densely shortly glandular-hairy, apically viscid	Supine or ascending, much branched, the lower part with densely downward white pilose, the upper part mixed glandular pilose	Erect or ascending, multibranched from woody base, glabrous or sparsely hairy, apically viscid
**Leaves**	Linear, lanceolate, or narrowly lanceolate, 2–3 cm long, 1.5–3.5 (–5) mm wide, both surfaces glabrous or pubescent, margins ciliate	Lanceolate, narrowly lanceolate or narrowly elliptic, 2–4.5 cm long, 0.5–1.2 cm wide, pilose on both surfaces, margin ciliate	Basal leaves patulate-oblanceolate; cauline leaves linear or linear-lanceolate, (1.5–) 3–5 (–6) cm long, 2–5 mm wide, glabrous
**Inflorescence**	Dichasial cymes 3–7-flowered	Dichasium often 3–15-flowered	Thyrse,lax, few-flowered
**Calyces**	Tubular-clavate, 1.2–1.5 cm long	Tubular, 3–3.3 cm long, ca. 0.4 cm in diam.	Tubular, 2.5–3.5 cm long, ca. 3 mm in diam.
**Androgynophore**	2–3 mm long, glabrous	1.5–1.8 cm long, glabrous	1.5–2.0 cm long, glabrous
**Petals**	Pale red	Pink	Pale red or white
**Limbs**	Obovate, 5–6 mm long, shallowly bifid	Narrowly obovate-oblong, 1.0–1.2 cm long, shallowly bifid	Obcordate or obovate, 1–1.4 cm, divided to about halfway
**Lobes of petals**	Nearly square, entire	Nearly square, entire or sometimes notched or lobed	Narrowly ovate, entire
**Capsules**	Ovoid, 0.6–0.8 cm long	narrowly ovate	Oblong, 1.0–1.5 cm long
**Seeds**	Reniform, seed ridge smooth	Orbicular-reniform, seed ridge smooth	Reniform, slightly flat, with a groove along the seed ridge, forming a shallow “V” shape

### ﻿Taxonomy

#### ﻿ Silene batangensis

Taxon classificationPlantaeCaryophyllalesCaryophyllaceae

Limpr. in Fedde, Repert. Sp. Nov. Beih. 12: 363 (1922).

2A053BC7-09BD-50FD-8024-483B8501C27D

≡ Melandriumbatangense (Limpr.) Pax et Hoffm. in Engler u. Prantl, Nat. Pflanzenfam. 2. Aufl. 16c: 343 (1934) —Lectotype (designated here): China, Sichuan Province, Batang County, on the way to Litang, on the rocky slopes, alt. 3400 m, *Limpricht H.W. n. 2232* (WU [WU0046563])— Fig. 1A.
= Silenedawoensis H. Limpr. in Fedde, Repert. Sp. Nov. Beih. 12: 363 (1922) syn. nov. — Lectotype (designated here): China, Sichuan Province, Dawu (Ressenyi), alt. 3450 m, July 1914, *Limpricht H.W. n.1966* (WU [WU0046562]) — Fig. 1B.

##### Description.

Herbs perennial. Roots clustered, fusiform. Stems supine or ascending, 20–35 cm long, multibranched, densely shortly glandular-hairy, apically viscid. Leaves linear, lanceolate, or narrowly lanceolate, 2–3 cm long, 1.5–3.5 (–5) mm wide, both surfaces glabrous or pubescent, margins ciliate, midvein prominent, base cuneate, apex obtuse or acute. Dichasial cymes 3–7-flowered, densely glandular hairy; flowers erect, 1.6–2 cm in diam. Pedicel 1–1.8 cm, slender, glandular hairy; bracts lanceolate, herbaceous. Calyx tubular-clavate, 1.2–1.5 cm long, densely pubescent, viscid, longitudinal veins violet; calyx teeth triangular-lanceolate, villous, margin membranous, apex obtuse. Androgynophore 2–3 mm long, glabrous. Petals included in calyx, pale red; claws oblanceolate, glabrous, auricles inconspicuous; limbs obovate, 5–6 mm long, shallowly bifid; lobes nearly square, entire; coronal scales orbicular. Stamens slightly exserted; filaments glabrous. Styles slightly exserted. Capsule ovoid, 6–8 mm. Seeds reniform, seed ridge smooth.

##### Distribution and habitat.

*Silenebatangensis* is distributed in western Sichuan and eastern Xizang, China. The species grows on sandy loam or alluvial soil of the forest margins or riparian shrub grasslands at elevations of 2500–3600 m.

##### Phenology.

Flowering from June to August and fruiting from August to October.

##### Additional specimens examined.

**China. Sichuan**: • Daofu County, roadside, 3200 m, 21 July 1974, *Sichuan Vegetation Team 05623* (CDBI); Daofu County, the hill behind the Martyr’s Cemetery, on the deserted slope, 3300 m, 29 June 1974, *B. C. Gao & Y.T.Wu 111649* (PE); Daofu County, 5 July 1977, *B. Z. Guo et al. 20904* (KUN, NAS); • Baiyu County, on the road from Baiyu to Dege, on slope, in thicket, 31°35'9.17"N, 98°38'43.95"E, 3176 m, 24 June 2017, *D. H. Liu 170165* (BNU); • Dege County, next to the Jinshajiang River Bridge, roadside, 3100 m, 31 July 1 *003330* (KUN, CDBI); • Derong County, Gajingxue Shan, 28°52'3"N, 99°14'45"E, alt. 3400 m, 19 July2004, *D.E.Boufford et al. 30843* (A); • Batang County, Songduo Country, beside National Highway G215, 30°15'40.56"N, 99°15'6.35"E, 2992 m, 6 August 2023, *F. Yang et al. BT22486* (YUKU); • Batang County, on the road from Batang to Baiyu, on mountain slope, scrub, 30°18'10.48"N, 99°16'00.54"E, 3118 m, 23 July 2010, *Kham Exp. 10-0465* (PE); • Batang County, the hill behind the military station, dry valley area, scrub, 2960 m, 22 June 1983, *Hengduan Mountains Vegetation Exp. 4255* (PE); • Batang County, Suwalong, the east bank of Jinshajiang River, river bank slope, in an arid thicket, 2600 m, 30 August 1981, *Qinghai-Tibet Exp. 005216* (HITBC, CDBI, KUN); • Batang County, on the road from Bakuang to Jinshajiang River, the bank of Bachu River, on sandy or gritty soil, in thicket, 2600 m, 21 July 1975, *C. L. Tang & Y. H. Guo 1817* (WUK); • Batang County, Xiaqiong Town, Xiaobachong, on the rocks in the valley, 3000 m, 22 July 1975, *C. L. Tang & Y. H. Guo 1836* (WUK); • Batang County, in thicket, 3400 m, 21 August 1982, *Q. Q. Wang 29409* (CDBI); • Batang County, 3308.55 m, 30 July 2014, *X. X. Zhu et al. CSH06571* (CSH); • Batang County, 29°45'35"N, 99°1'28"E, 3276.9 m, 29 July 2014, *X. X. Zhu et al. CSH06577* (CSH); Batang County, 3222.72 m, 29 July 2014, *Bin Chen et al. CSH06708* (CSH); • Daocheng County, Mengzi, Wage village, growing among shrubs on sunny slope, 2900 m, 17 August 1973, *Sichuan Vegetation Team 2503* (CDBI, PE); Daocheng County, in thicket, 2500 m, 18 June 1971, *Sichuan Vegetation Team 0039* (CDBI). **Xizang**: • Changdu County, South of the city of Changdu, on road (highway 214) to Bangda, along the Langcangjiang (Mekong River), xeric slope and adjacent ravine running to the river, xeric shrub and herb vegetation dominant, growing among shrubs on dry steep slope, 30°51'33"N, 97°20'24"E, 3080 m, 20 July 2000, *D. E. Boufford et al. 29562* (PE); Chaya County, near Big footprints, along the Langcangjiang, 30°53'58.46"N, 97°21'42.56"E, 3117 m, 13 August 2023, *F. Yang et al. CY22880* (YUKU); • Chaya County, near Jitang Town, roadside, in mountainous region, 3000 m, September 1976, *Qinghai-Tibet Supplemental Exp. 5999* (KUN); • Chaya County, Jitang Town, Youxi, near the ditch, 3600 m, 8 July 1976, *Qinghai-Tibet Exp. 12335* (KUN); • Gongjue County, Doba village ca. 13.9 km in a straight line northeast of the city of Gongjue, along the Re Qu, a tributary of the Jinshajiang River, dry slope, 30°58'37"N, 98°19'49"E, 3510-3550 m, 31 July 2009, *D. E. Boufford et al. 41523* (P, KUN); Jiangda County, east of the city of Jiangda, Tong Pu Xiang, Jiba Cun on road to Dege County, in Slopes along Du Qu and adjacent roadside, among stones, 31°34'11"N, 98°18'31"E, alt. 3386 m, 30 July 2004, *D. E. Boufford et al. 31362* (KUN); • Mangkang County, near Rumei village, by the Lancang River, 29°40'7.66"N, 98°22'26.15"E, alt. 2970 m, 15 August 2023, *F. Yang et al. MK22969* (YUKU); • Mangkang County, Zhubalong village, Erdaoban, in the bushes, 29°41'42.10"N, 98°55'20.18"E, alt. 2762 m, 5 August 2024, *F. Yang et al. MK26730* (YUKU).

#### ﻿ Silene longiuscula

Taxon classificationPlantaeCaryophyllalesCaryophyllaceae

C. Y. Wu et C. L. Tang in Fl. Yunnan. 6: 236. Addenda 837 (1995).

2604B018-7B9A-5EB2-9342-1151DFCA9678

##### Type.

China • Yunnan Province, Yuanmou County, Jiangbian, Longjie village, Sangangpo, scrubs, alt. 1600 m, 7 August 1965, *Pl. Oleifer. Exp. 65-709* (Holotype: KUN [KUN1205497]; isotypes: LBG [LBG00055690], KUN [KUN1205500]). — Fig. 1C.

##### Description.

Herbs perennial. Stems supine or ascending, much branched, the lower part with densely downward white pilose, the upper part mixed glandular pilose. Leaves numerous, lanceolate, narrowly lanceolate or narrowly elliptic, 2–4.5 cm long, 0.5–1.2 cm wide, base cuneate or broadly cuneate, apex acute, with long mucronulate, pilose on both surfaces, margin ciliate, midribs slightly concave adaxially, raised abaxially, with densely pilose, lateral veins 1–2 pairs, slender; petiole short, broad, 1–3 mm long, with densely ciliate. Dichasium often 3–15-flowered; bracts leaflike, linear-lanceolate to subulate, 0.3–0.6 cm long, margin and midribs with densely glandular pilose. Pedicel erect or slightly bending, 2–3.5 cm long, with densely glandular pilose. Calyx tubular, 3–3.3 cm long, ca. 0.4 cm in diam., calyx teeth ovate-triangular, apex round or obtuse, margin membranous, densely ciliate, longitudinal veins 10, violet, not converging at apex, densely glandular pilose. Androgynophore 1.5–1.8 cm long, glabrous. Petals pink, 2.8–3.0 cm long, claws cuneate, exserted beyond calyx, not auriculate, glabrous at base; limbs narrowly obovate-oblong, 1.0–1.2 cm long, shallowly bifid; lobes nearly square, entire or sometimes notched or lobed, with one linear tooth on each lateral side, ca. 1.5 mm long; coronal scales ca. 1 mm long, entire. Stamens 10, subequal to claws, exserted beyond calyx; anther oblong, ca. 1.5 mm long, filaments glabrous; ovary narrowly elliptical, 0.8–1.0 cm long, styles 3, exserted beyond calyx. Capsule narrowly ovate, exserted beyond persistent calyx, dehiscing with 6; seeds orbicular-reniform, ca. 1 mm long, seed ridge smooth.

##### Distribution and habitat.

*Silenelongiuscula* appears to be a very rare species, known only from three collections from Yunnan and Sichuan. It grows in riparian or hillside shrub grasslands at elevations of 1200–1800 m.

##### Phenology.

Flowering and fruiting times from July to November.

##### Additional specimens examined.

**China Sichuan**: • Miyi County, in riparian shrub grasslands, alt. 1250 m, 19 November 1982, *D. Liu 23962* (CDBI); • Yanbian County, Taitian village, at roadside, 19 November 1978, *Yanbian Exp. 630* (SM). **Yunnan**: • Yuanmou County, Longjie village, beside the Jinshajiang River, sunny hillside, scrub grasslands, alt. 1800 m, 14 September 1963, *Ging-Sa-Kiang Exp. 63-6900* (KUN, PE).

#### ﻿ Silene markamensis

Taxon classificationPlantaeCaryophyllalesCaryophyllaceae

L. H. Zhou in Fl. Xizang. 1: 734 (1983).

875C0409-2901-5605-B547-89B8A0F74A6A

##### Type.

China • Xizang, Mangkang County, on the road from Mangkang to Batang, 3000 m, 9 September 1977, *B. Z. Guo and W. Y. Wang 23584* (Holotype: HQ, Isotypes: HNWP [HNWP68194, HNWP187674, HNWP183901]). — Fig. 1D.

##### Description.

Herbs perennial, 30–80 cm long. Roots robust, conical, lignified. Stems erect or ascending, multibranched from woody base, glabrous or sparsely hairy, apically viscid. Basal leaves patulate-oblanceolate, withered at anthesis; cauline leaves with short sterile axillary branches, linear or linear-lanceolate, (1.5–) 3–5 (–6) cm long, 2–5 mm wide, base cuneate or attenuate, glabrous, apex acuminate, midvein prominent. Flowers erect, in a lax, few-flowered thyrse; cymules with peduncles subequaling pedicels, opposite or alternate, often 1-flowered. Pedicel 0.5–4 cm long, slender, glabrous, viscid; bracts linear, 5–10 mm long, ciliate. Calyx tubular, clavate in fruit, pale yellow, 2.5–3.5 cm long, ca. 3 mm in diam., glabrous, veins green or violet; calyx teeth ca. 1.5 mm. Androgynophore 1.5–2.0 cm long, glabrous. Petal claws slightly exserted beyond calyx, narrowly lanceolate, 1–1.5 cm, glabrous, not auriculate, limbs pale red or white, obcordate or obovate, 1–1.4 cm, divided to about halfway; lobes narrowly ovate, entire; coronal scales very small. Stamens and styles exserted; filaments glabrous. Ovary oblong, ca. 1 cm long; styles 3, ca. 1.3 cm long. Capsule oblong, 1.0–1.5 cm long. Seeds dark brown, reniform, slightly flat, ca. 1.2 mm, with a groove along the ridge, forming a shallow “V” shape.

##### Phenology.

*Silenemarkamensis* was observed flowering from June to August and fruiting from August to September.

##### Distribution and habitat.

This species is endemic to Xizang and Sichuan of China. It grows in grasslands, cliffs, at 1400–3600 m.

##### Notes.

The distribution in Yunnan is doubtful, and the specimen (Deqen, *C. Y. Wu 4409* (KUN)) cited by “Flora Reipublicae Popularis Sinicae” is actually a misidentification of *Sileneesquamata*. *Silenemarkamensis* is superficially similar to *S.esquamata* but distinct mostly by its basal leaves oblanceolate (vs. spatulate-oblanceolate), cauline leaves linear or linear-lanceolate (vs. broadly oblanceolate), calyx tubular, 2.5–3.5 cm (vs. 1.2–1.5 cm) long, androgynophore 1.5–2.0 cm (vs. 0.5 cm) long, limbs white or pale red (vs. pink).

##### Additional specimens examined.

**China. Sichuan**: • Batang County, Zhubalong village, east bank of Jinshajiang River Valley, hillside thickets, 2450 m, 29 August 1981, *Qinghai-Tibet Team 005195* (KUN, CDBI, HITBC); • Litang County, on rocky, 2000 m, 9 July 1930, *F. T. Wang 21682* (KUN, PE, WUK); • Litang County, on rocky, 1800 m, 18 August 1933, *T. T. Yu 2493* (PE, CQNM, IBSC, LBG); • Xiangchen County, sunny and grassy slope, 3100 m, 30 September 1973, *Sichuan Vegetation Team 3567* (CDBI, KUN, PE). **Xizang**: • Mangkang County, ca. 9 KM on G318 road from Rumei to Zuogong, roadside, on the valley slope, 29°34'38"N, 98°18'50"E, 3103 m, 13 September 2008, *T. Zhang et al. 08CS677* (KUN); • Mangkang County, Zhubalong village, near Xiquhe Bridge, dry-hot valley scrubs, 29°45'20.07"N, 98°57'37.33"E, 2525–2560 m, 19 August 2010, *Kham Expedition 10–2318* (PE); • Mangkang County, Zhubalong village, on the way from Sandaoban to Erdaoban, along National Highway G318, 29°41'51.8"N, 98°52'26.52"E, 2912 m, 15 August 2023, *F. Yang et al. MK22974* (YUKU).

## Supplementary Material

XML Treatment for Silene batangensis

XML Treatment for Silene longiuscula

XML Treatment for Silene markamensis

## ﻿Additional information

### Author ORCIDs

Huan-Chong Wang https://orcid.org/0000-0001-8562-8849

### Data availability

All of the data that support the findings of this study are available in the main text.
